# Attenuation of Skeletal Muscle and Renal Injury to the Lower Limb following Ischemia-Reperfusion Using mPTP Inhibitor NIM-811

**DOI:** 10.1371/journal.pone.0101067

**Published:** 2014-06-26

**Authors:** David Garbaisz, Zsolt Turoczi, Peter Aranyi, Andras Fulop, Oliver Rosero, Edit Hermesz, Agnes Ferencz, Gabor Lotz, Laszlo Harsanyi, Attila Szijarto

**Affiliations:** 1 Semmelweis University, 1^st^ Department of Surgery, Budapest, Hungary; 2 University of Szeged, Department of Biochemistry and Molecular Biology, Szeged, Hungary; 3 Semmelweis University, 2^nd^ Department of Pathology, Budapest, Hungary; Georgia Regents University, United States of America

## Abstract

**Introduction:**

Operation on the infrarenal aorta and large arteries of the lower extremities may cause rhabdomyolysis of the skeletal muscle, which in turn may induce remote kidney injury. NIM-811 (N-metyl-4-isoleucine-cyclosporine) is a mitochondria specific drug, which can prevent ischemic-reperfusion (IR) injury, by inhibiting mitochondrial permeability transition pores (mPTP).

**Objectives:**

Our aim was to reduce damages in the skeletal muscle and the kidney after IR of the lower limb with NIM-811.

**Materials and methods:**

Wistar rats underwent 180 minutes of bilateral lower limb ischemia and 240 minutes of reperfusion. Four animal groups were formed called Sham (receiving vehicle and sham surgery), NIM-Sham (receiving NIM-811 and sham surgery), IR (receiving vehicle and surgery), and NIM-IR (receiving NIM-811 and surgery). Serum, urine and histological samples were taken at the end of reperfusion. NADH-tetrazolium staining, muscle Wet/Dry (W/D) ratio calculations, laser Doppler-flowmetry (LDF) and mean arterial pressure (MAP) monitoring were performed. Renal peroxynitrite concentration, serum TNF-α and IL-6 levels were measured.

**Results:**

Less significant histopathological changes were observable in the NIM-IR group as compared with the IR group. Serum K^+^ and necroenzyme levels were significantly lower in the NIM-IR group than in the IR group (LDH: p<0.001; CK: p<0.001; K^+^: p = 0.017). Muscle mitochondrial viability proved to be significantly higher (p = 0.001) and renal function parameters were significantly better (creatinine: p = 0.016; FENa: p<0.001) in the NIM-IR group in comparison to the IR group. Serum TNF-α and IL-6 levels were significantly lower (TNF-α: p = 0.003, IL-6: p = 0.040) as well as W/D ratio and peroxynitrite concentration were significantly lower (p = 0.014; p<0.001) in the NIM-IR group than in the IR group.

**Conclusion:**

NIM-811 could have the potential of reducing rhabdomyolysis and impairment of the kidney after lower limb IR injury.

## Introduction

Arterial occlusive diseases are the most frequent causes of acute limb ischemia (ALI). Operative treatments for these diseases, as well as other reconstructive surgeries on abdominal aorta or other major arteries of the lower limb may induce sudden onset ischemia, in which event lower limb tissues suffer ischemic-reperfusion (IR) injuries.

There are two sides to IR injury since, in a paradox manner, reestablishment of the blood supply is associated with further damages. Beyond local muscle cell necrosis and consequent rhabdomyolysis, serious remote organ injuries may also develop. The metabolites and proinflammatory cytokines released from the damaged muscles [1]–[3] may induce systemic inflammatory response syndrome (SIRS), which could possibly affect the gastrointestinal system, lungs and kidneys, thereby inducing multiple organ dysfunction syndrome (MODS).

Mitochondria play a leading role in IR injury [4]. Damage caused by mitochondrial membrane depolarization can significantly jeopardize energy production, which can lead to cell damage, then further to cell death. The mechanism of mitochondrial membrane depolarization, called mitochondrial permeability transition (MPT), is induced by the opening of the mitochondrial permeability transition pores (mPTP) in the mitochondrial membrane [5]. Several proteins participate in the structure of the pore, but the most important during mPTP opening is cyclophilin-D in the mitochondrial matrix [6], [7]. After opening of the pore, the mitochondrial electron transport chain is interrupted, which disarrays mitochondrial energy production and induces production of reactive oxygen species (ROS) [6]. Decline of the mitochondrial membrane potential causes further opening of the mPTPs, thus release of different molecules from the dysfunctional mitochondria may provoke cell death [6].

Cyclosporine-A (CsA) – besides its known immunosuppressive effects – is a potential inhibitor of the opening of mPTP. It can bind to both the cyclophilin-D component of mPTPs and the cytosolic cyclophilin-A molecules [8]–[11]. N-methyl-4-isoleucine cyclosporine (NIM-811) is a derivative of CsA, which does not bind to cyclophilin-A, therefore it has no immunosuppressive effects; it does however inhibit the opening of mPTP by binding to cyclophilin-D, thus preventing the development of MPT [12]. NIM-811 has an enormous advantage over CsA in that it has no known systemic side effects.

Based on our earlier investigations, attenuation of local IR damage can reduce the risk of remote organ injury (i. e. in the kidneys) [13]–[15]. During IR, opening of the mPTP plays a pivotal role in cell injury, thus inhibition of the pore opening may mitigate the damages. In the current study therefore, our aim was to examine the potentially favourable effects of NIM-811 as an inhibitor of mPTP opening in a skeletal muscle model of IR injury, with respect to local damages, consequential remote organ complications, as well as renal dysfunction.

## Materials and Methods

Male Wistar rats (n = 40) weighing 220–250 g were used (Charles Rivers Hungary Ltd., Budapest, Hungary). Animals were kept under specific pathogen-free conditions at 22–24°C, on standard rat chow and water ‘ad libitum’. The experimental design was carried out in strict accordance with the recommendations in the Guide for the Care and Use of Laboratory Animals of the National Institutes of Health (The National Academies Press, Washington, D.C., USA, 8th edition). The protocol was approved by the Committee on Animal Experimentation of Semmelweis University (Permit Number: 22.1/2409/3/2011). All surgeries were performed under general anesthesia with efforts to minimize suffering.

### Preoperative procedures

Animals were anesthetized by intraperitoneal injection of 75 mg/bwkg ketamine and 7.5 mg/bwkg xylazine. Anesthesia was maintained by intravenous (through right jugular vein) administration of 25 mg/bwkg/h ketamine and 2.5 mg/bwkg/h xylazine. Heating blankets were used to keep body temperature at 37°C ±0.5°C, monitored with rectal probe (Homeothermic Blanket Control Unit, Harvard Apparatus, Holliston, MA, USA).

Mean arterial pressure (MAP) was registered by invasive blood pressure monitor (Kent Scientific Corporation, Torrington, CT, USA) through the internal carotid artery.

### Surgical procedures and experimental design

Before induction of ischemia, a 20-minute baseline period was allowed. Tourniquet was applied around both lower limbs in the femoral region [16], and rats underwent a 180-minute long total bilateral lower limb ischemia. After median laparotomy, a laser-Doppler flowmeter (LDF) probe was placed over the front surface of the left kidney in order to monitor circulation of the renal parenchyma [17]. Following a 1 cm longitudinal skin incision along the lateral side of the thigh, another LDF probe was placed on the femoral biceps muscle to monitor the microcirculation of the left lower limb [14].

The animals were randomized to the following four groups according to treatment: Sham (receiving vehicle and sham surgery, n = 10), NIM-Sham (receiving NIM-811 and sham surgery, n = 10), IR (receiving vehicle and surgery, n = 10) and NIM-IR (receiving NIM-811 and surgery, n = 10). Five minutes before reperfusion, NIM-811 (10 mg/bwkg; Novartis International AG, Basel, Switzerland) was administered intravenously to the NIM-Sham and NIM-IR groups. After removal of the tourniquet, reperfusion was allowed for 240 minutes. Sham animals received all procedures including anesthesia and laparotomy except for the 180-minute-long ischemia.

During the experiment, urine output was determined by gathering the spontaneously excreted urine in two fractions: (1) urine samples collected for 20 minutes before the ischemia served as control; (2) the amount of urine gathered during the last 3 hours of reperfusion was completed with bladder puncture at termination of the experiment.

### Determining skeletal muscle injury

#### Serum laboratory parameters

Tests consisted of measuring serum creatine kinase (CK), lactate dehydrogenase (LDH) and K^+^ levels. After centrifuging (2×10 min, 1050 g) the blood samples, serum was snap-frozen in liquid nitrogen and stored at −80°C until analysed with a clinical chemistry analyzer automate (Beckman Coulter AU480/2011, Beckman Coulter Inc, Brea, CA, USA).

#### Histological evaluation of skeletal muscle

Samples were collected from the anterior tibial muscle and fixed in 4% neutral buffered formalin solution for one day. Thereafter samples were embedded in paraffin, 3 µm thin cross- and longitudinal sections were cut and stained for hematoxillin and eosin. Histological examinations were carried out with Olympus BX50 microscope equipped with Olympus DP70 camera (Olympus Corporation, Tokyo, Japan). The examining pathologist received no informed about the applied pretreatment.

#### Assessment of muscle fiber viability

Samples from the left anterior tibial muscle were snap frozen in liquid nitrogen and stored at −80°C until further processing. Cross sections of 3 µm thickness were cut in cryostat. Slides were incubated for 30 minutes at 37°C in a solution consisting of nitroblue tetrazolium (1.8 mg/dL), NADH (15 mg/dL) and 0.05 M TRIS buffer (pH 7.6) (Sigma-Aldrich Inc, St. Louis, MO, USA). Unused tetrazolium reagent was removed using ascending and descending concentrations of acetone. Ten different fields were photographed randomly in each slide with 600× magnification. Viability assessment was performed by detection of NADH-tetrazolium reductase staining of muscles using Leica QWin Pro (Leica Microsystems Ltd, Wetzlar, Germany) morphometric software. Muscle fiber viability was calculated as a proportion of the total stained area and the total muscle fiber area of the slide. Average of the 10 slides was calculated for each animal. The final result is expressed as a percentage of the values of untreated control muscles (obtained in a previous experiment) [18].

#### Skeletal muscle Wet/Dry (W/D) ratio

Tissue edema was quantified with the wet/dry ratio utilizing the remaining tibial anterior muscle. Following careful excision, muscles were weighed immediately after the end of reperfusion (wet weight) and placed in a drying oven set at a temperature of +80°C until reaching constant weight. Muscles were then reweighed (dry weight) [19] and wet/dry ratio calculated using the following equation: (wet weight – dry weight)/wet weight * 100.

#### Measurement of tissue microcirculation

Registration of microcirculation of the lower limb skeletal muscle was performed by laser Doppler flowmeter (MOOR Instruments Ltd, London, UK; DRT4). The probe of the device was placed on the surface of the left femoral biceps muscle. To characterize the microcirculation, reperfusion area (RA) was used, based on the mathematical calculations of our research group. The mathematical transformations required for correct interpretation of the circulation data were described previously [20].

### Systemic inflammatory parameters

#### Measurement of TNF-α and IL-6 levels

Proinflammatory cytokine concentrations were measured by sandwich ELISA kits according to manufacturer’s instructions (R&D Systems, Minneapolis, MN, USA). Absorbance was measured at 450nm.

### Determination of kidney dysfunction

#### Histopathology

Tissue sampling was performed from the same anatomical location (left kidney) in case of every animal, regardless of group. As in case of skeletal muscle samples, kidney samples were fixed in 4% neutral buffered formalin solution (24 h), followed by embedment in paraffin, then 3 µm thin sections were cut and stained for hematoxillin and eosin. During evaluation of the sections (Olympus DP70 camera, Olympus Corporation, Tokyo, Japan), features of tubular and interstitial injuries were considered.

Evaluation of histological damage was performed in keeping with a score described previously [21]. The degree of tubular damage to the kidney tissue was scored by determining the percentage of tubules in the kidney cortex, which showed tubular damage, tubular cell necrosis and cast formation as follows: 0, none; 1≤10%; 2, 10–25%; 3, 25–45%; 4, 45–75%; 5>75%. Ten randomly chosen, non-overlapping fields per section were examined. Scoring was carried out by an independent pathologist in a blinded manner.

#### Laboratory examinations

Blood samples were investigated according to the same method as serum necroenzymes. Levels of Na^+^-, creatinine- and BUN (blood urea nitrogen) were determined, from which renal injury parameters (BUN/creatinine ratio, fractional Na^+^-excretion (FENa = U_Na+_×P_kreat._ x100/U_kreat._×P_Na+_) were calculated.

#### Registration of renal microcirculation

During the experiment, microcirculation of the left kidney cortex was registered with LDF, placing the laser probe on the anterior surface of the left kidney.

#### Measurement of peroxynitrite concentrations in the kidneys

Homogenized samples of kidney tissue were analyzed by spectrophotometric method after dilution in 1.0 M NaOH (60∶1) solution; absorbance was measured at 302 nm. As control, 100 mM potassium-phosphate (pH 7.4) was added to the sample (60∶1). The rate of decline in absorbance was measured on neutral pH [22].

### Biochemicals

NIM-811 was the generous gift of Novartis International AG (Basel, Switzerland). The agent (10 mg/bwkg) was dissolved in the vehicle, which contained 1.3 mL cremophor oil (Cremophor EL, polyethoxylated castor oil), 0.7 mL ethanol and 8 mL 0.9% saline solution [23]. The injection volume range was standard (0.44–0.5 mL) in each animal according to their weights (220–250 g). Other reagents were purchased from Sigma-Aldrich Corporation (St Louis, MO, USA).

### Statistical analysis

Data are shown as means ± SEM. Statistical analysis of data was performed using IBM SPSS Statistics 20.0 software (IBM Corporation, Armonk, NY, USA). Two-way analysis of variance (ANOVA) was used for comparison of all groups with LSD post-hoc tests. A 95% confidence interval was considered as statistically significant (p<0.05).

## Results

### Determining skeletal muscle injury

#### Histopathology

**Figure 1:**
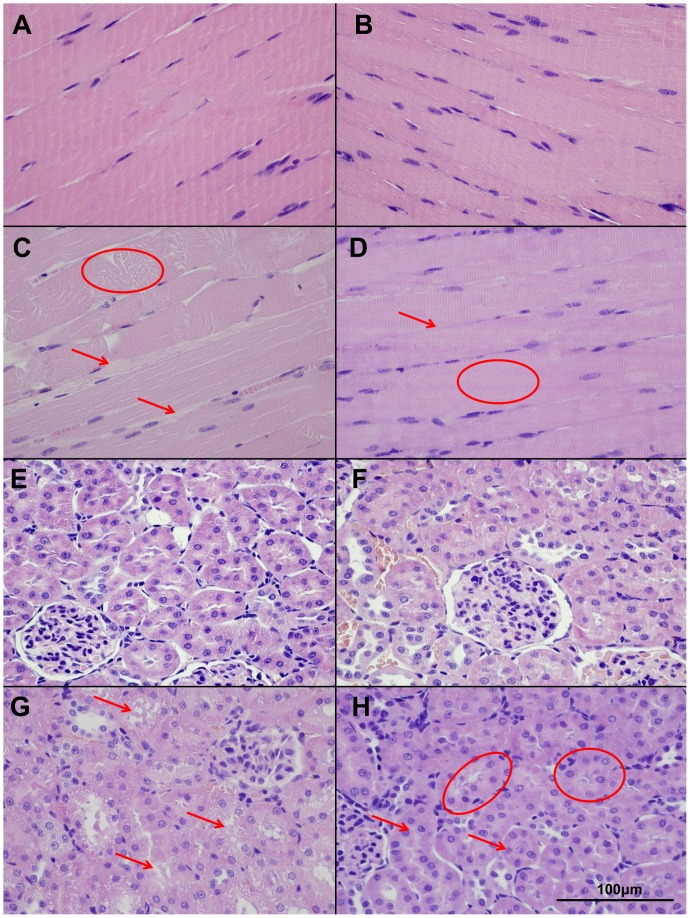
Muscle and kidney histopathology (hematoxillin-eosin (HE) stain, light microscopy). (**A–B**)**:** An almost physiologically histological picture is observable in the Sham (A) and NIM-Sham (B) groups, with intact muscle fibers and normal wide interstitial spaces. (**C**)**:** In the IR group segmental necrosis (circle), disintegrated myofilaments and thicker interstitial spaces (arrow) are detectable. (**D**)**:** NIM-IR group shows intact muscle fibers (circle) with no expanded interstitial spaces (arrow), similar to the normal structure. (**E–F**)**:** Sham and NIM-Sham groups show the normal structure of kidney cortical tissue. Tubules have normal appearance. (**G**)**:** In the IR group massive injury can be detected with loss of cell integrity and intracellular vacuolization (arrows). (**H**)**:** In the NIM-IR group cell necrosis is less severe (arrow) and the tubular integrity remained intact (circle), an almost normal picture can be seen.

As compared with the nearly normal histological picture detectable in case of the Sham and NIM-Sham groups, segmental necroses and disintegrated filaments were observable in the muscle fibers in case of the IR group. The muscle fibers were separated by significant edema. By contrast, in the NIM-IR group, intact conditions were observable (Figure 1 A–D).

#### Laboratory measurements

**Figure 2:**
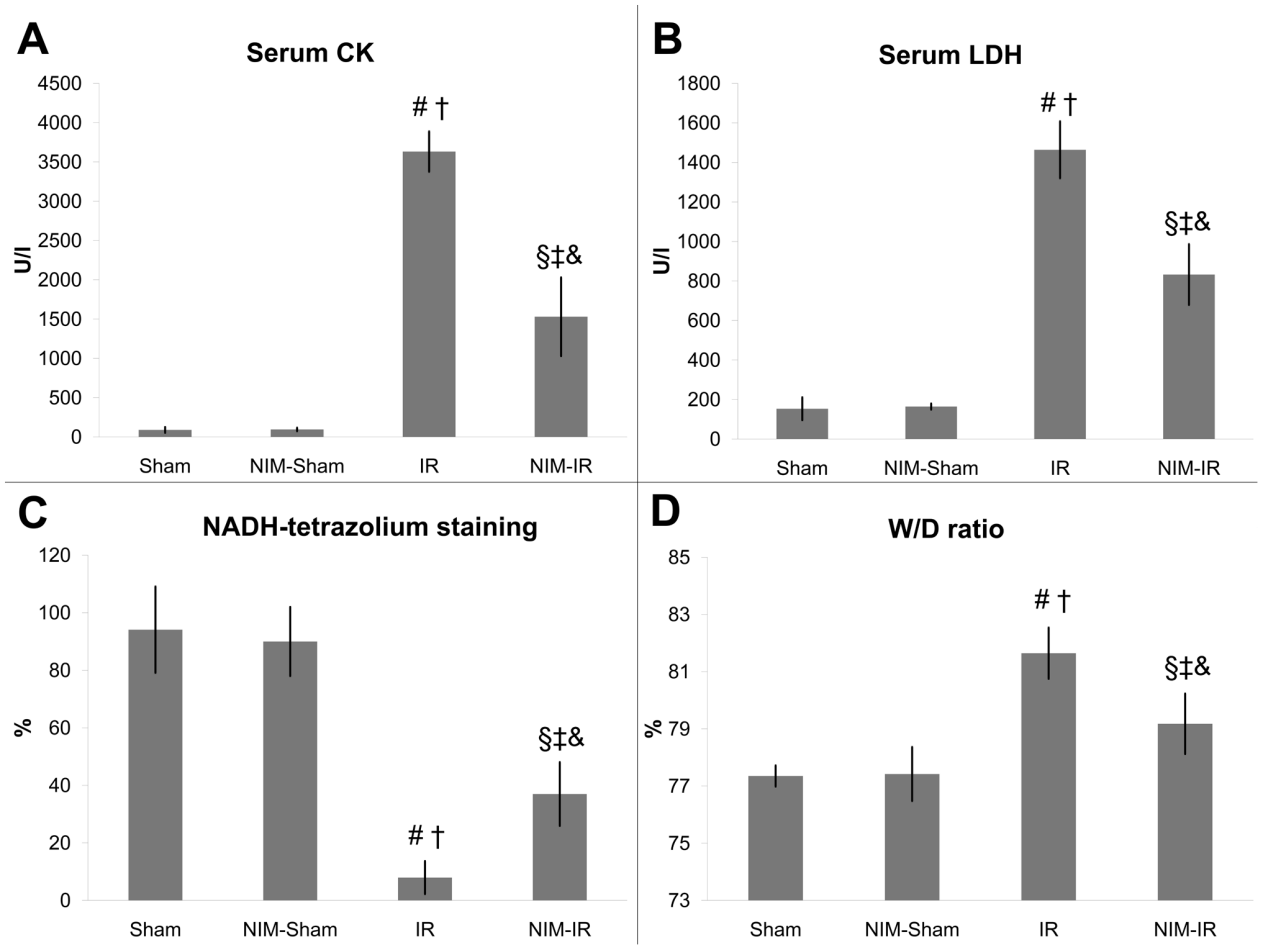
Parameters of muscle injury, muscle fiber viability and muscle wet content. (**A–B**)**:** Serum creatine-kinase (CK) and lactate-dehydrogenase (LDH) concentrations were significantly elevated in the IR group compared with the Sham and NIM-Sham groups. Significantly lower value was detectable in the NIM-IR group compared with the IR group, leading to the conclusion that there may be a lower extent of muscle necrosis (CK: # p<0.01 vs. Sham; † p<0.01 vs. NIM-Sham; § p<0.01 vs. IR; ‡ p<0.01 vs. Sham; & p<0.01 vs. NIM-Sham; LDH: # p<0.01 vs. Sham; † p<0.01 vs. NIM-Sham; § p<0.01 vs. IR; ‡ p<0.01 vs. Sham; & p<0.01 vs. NIM-Sham; U/l – Unit/liter). (**C**)**:** Muscle fiber viability was assessed by NADH-tetrazolium staining and was expressed as percentage of viability measured in untreated controls (%). In the IR group there was a significant decline in viability compared with the Sham and NIM-Sham groups. Significantly higher value was found in the NIM-IR group, compared with the IR group (# p<0.01 vs. Sham; † p<0.01 vs. NIM-Sham; § p<0.01 vs. IR; ‡ p<0.01 vs. Sham; & p<0.05 vs. NIM-Sham). (**D**)**:** Wet/Dry ratio is suitable to determine the amount of interstitial edema. The wet content of the skeletal muscle tissue was significantly lower in the NIM-IR group compared with the IR group (# p<0.01 vs. Sham; † p<0.01 vs. NIM-Sham; § p<0.05 vs. IR; ‡ p<0.05 vs. Sham; & p<0.05 vs. NIM-Sham).

Both CK and LDH levels were significantly increased in the IR group compared with the Sham and NIM-Sham groups (values were almost similar in the latter two groups). Both examined parameters were significantly lower in the NIM-IR group than in the IR group (CK: p<0.001; LDH: p<0.001). The serum K^+^ level was higher in the IR group compared with the Sham and NIM-Sham groups, and the elevation was significantly lower in the NIM-IR group (p = 0.017) (Figure 2 A–B, Table 1).

**Table 1:** Laboratory measurements, haemodynamics data and wet/dry ratios.

		Sham	NIM-Sham	IR	NIM-IR	P-value(IR vs. NIM-IR)
**Parameters** **of muscle** **injury**	LDH (U/l)	153.40±57.60	164.40±15.19	1464.0±143.82	832.5±153.78	P<0.001
	CK (U/l)	90.0±36.24	95.60±22.05	3632.22±256.66	1530.33±500.0	P<0.001
	K^+^ (mmol/l)	4.0±0.56	4.20±0.65	7.79±0.96	6.53±1.05	P = 0.017
	Muscle fiber viability (%)	94.10±15.0	90.0±12.0	7.94±5.71	36.97±11.06	P = 0.001
	W/D (%)	77.35±0.36	77.41±0.91	81.64±0.89	79.17±1.05	P = 0.014
	LDF RA (%)	101.23±2.63	91.98±2.47	22.81±14.86	45.43±23.74	P<0.001
**Systemic** **parameters**	TNF-α (pg/ml)	15.0±5.0	20.0±8.0	172.142±25.0	55.34±17.69	P = 0.003
	IL-6 (pg/ml)	110.0±25.0	125.0±32.0	563.29±120.0	224.73±107.13	P = 0.040
	MAP (during reperfusion; Hgmm)	102.17±0.66	102.32±3.67	88.04±7.22	97.83±11.47	P = 0.044
**Parameters** **of remote** **kidney** **injury**	Histopathological score	1.08±0.20	1.33±0.25	4.0±0.81	2.64±0.37	P<0.001
	Urine output (during reperfusion; ml/h)	1.0±0.8	0.95±0.10	0.09±0.04	0.27±0.07	P = 0.022
	Creatinine (µmol/l)	79.14±47.23	83.94±19.37	150.17±42.91	96.95±32.21	P = 0.001
	BUN/creatinine	0.09±0.01	0.09±0.01	0.07±0.01	0.09±0.01	N.S.
	FENa (%)	0.21±0.10	0.22±0.12	1.27±0.32	0.32±0.15	P<0.001
	LDF RA (%)	97.90±1.76	90.85±4.73	86.67±15.63	83.01±16.93	N.S.
	Peroxynitrite (nmol/mg protein)	0.15±0.03	0.16±0.01	0.24±0.02	0.14±0.03	P<0.001

LDH: lactate dehydrogenase; CK: creatine kinase; W/D: wet/dry ratio; LDF RA: Laser Doppler flowmeter, reperfusion area; TNF-α: tumor necrosis factor alpha; IL-6: interleukin 6; MAP: mean arterial pressure; BUN: blood urea nitrogen; FENa: fractional Na^+^ excretion.

#### Muscle fiber viability

In the IR group, muscle fiber viability was significantly decreased as compared with the Sham and NIM-Sham groups (where values were nearly 100 percent). Viability in the NIM-IR group was significantly less reduced in comparison with the IR group (p = 0.001) (Figure 2C, Table 1).

#### Wet/Dry ratio of skeletal muscle tissues

Calculations of wet content of muscles revealed a significantly higher value in the IR group as compared with the Sham and NIM-Sham groups. In the NIM-IR group, the amount of tissue edema was significantly lower than in the IR group (p = 0.014) (Figure 2D, Table 1).

#### Microcirculation of skeletal muscle of the lower limb

**Figure 3:**
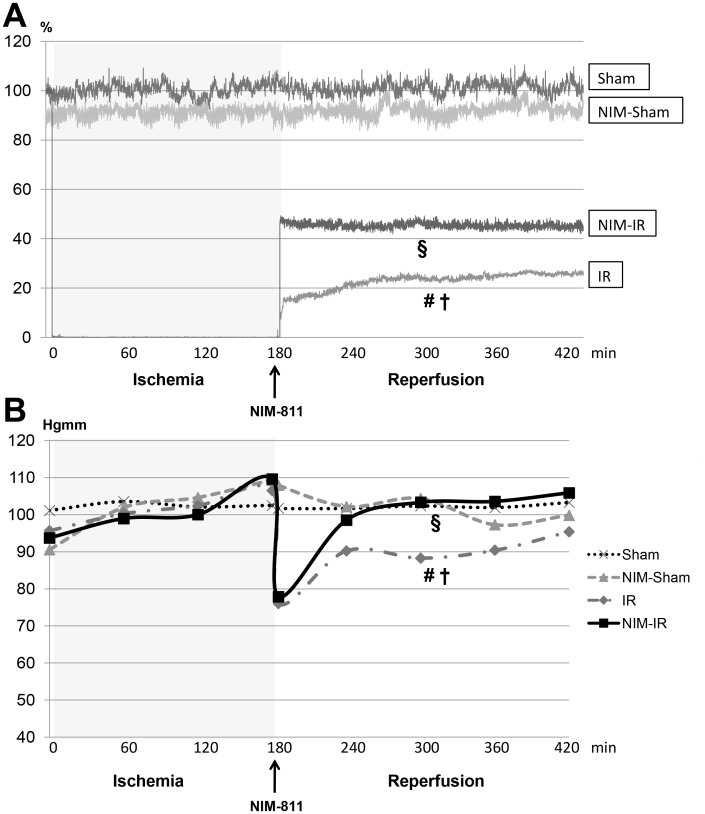
Systemic hemodinamycs and microcirculation of skeletal muscle of lower limb. (**A**)**:** Microcirculation of the lower limb skeletal muscle was monitored by laser Doppler flowmeter (LDF). Data are shown as percentage of baseline flow before ischemia (%). In the IR group a significant decline can be observed compared with the Sham and NIM-Sham groups after the onset of reperfusion. Microcirculation became stabilized at a significantly higher level in the NIM-IR group than in the IR group (# p<0.05 vs. Sham; † p<0.05 vs. NIM-Sham; § p<0.05 vs. IR). (**B**)**:** Mean arterial pressure (MAP) was registered during blood pressure monitoring. MAP of the Sham and NIM-Sham groups remained constant during the entire experimental period, whereas values of both the IR and NIM-IR groups decreased at the beginning of reperfusion. MAP of the NIM-IR group was significantly higher after the onset of reperfusion as compared with the IR group (# p<0.05 vs. Sham; † p<0.05 vs. NIM-Sham; § p<0.05 vs. IR).

Calculations of the reperfusion areas displayed significantly compromised microcirculation observable in the IR group when compared with the Sham and NIM-Sham groups. Microcirculation became stabilized at a significantly higher level in the NIM-IR group than in the IR group (RA: p<0.001) (Figure 3A, Table 1).

### Systemic parameters

#### Systemic hemodynamics

MAP values in case of the Sham and NIM-Sham groups remained at the same level during the entire experimental period. In case of both the IR and NIM-IR group, the MAP values showed a decline by 30 and 32 mmHg, respectively at the beginning of the reperfusion. After the beginning of the reperfusion the MAP values were found to be significantly higher in the NIM-IR group compared with the IR group (p = 0.044) (Figure 3B, Table 1).

#### Measurement of TNF-α and IL-6 levels

**Figure 4:**
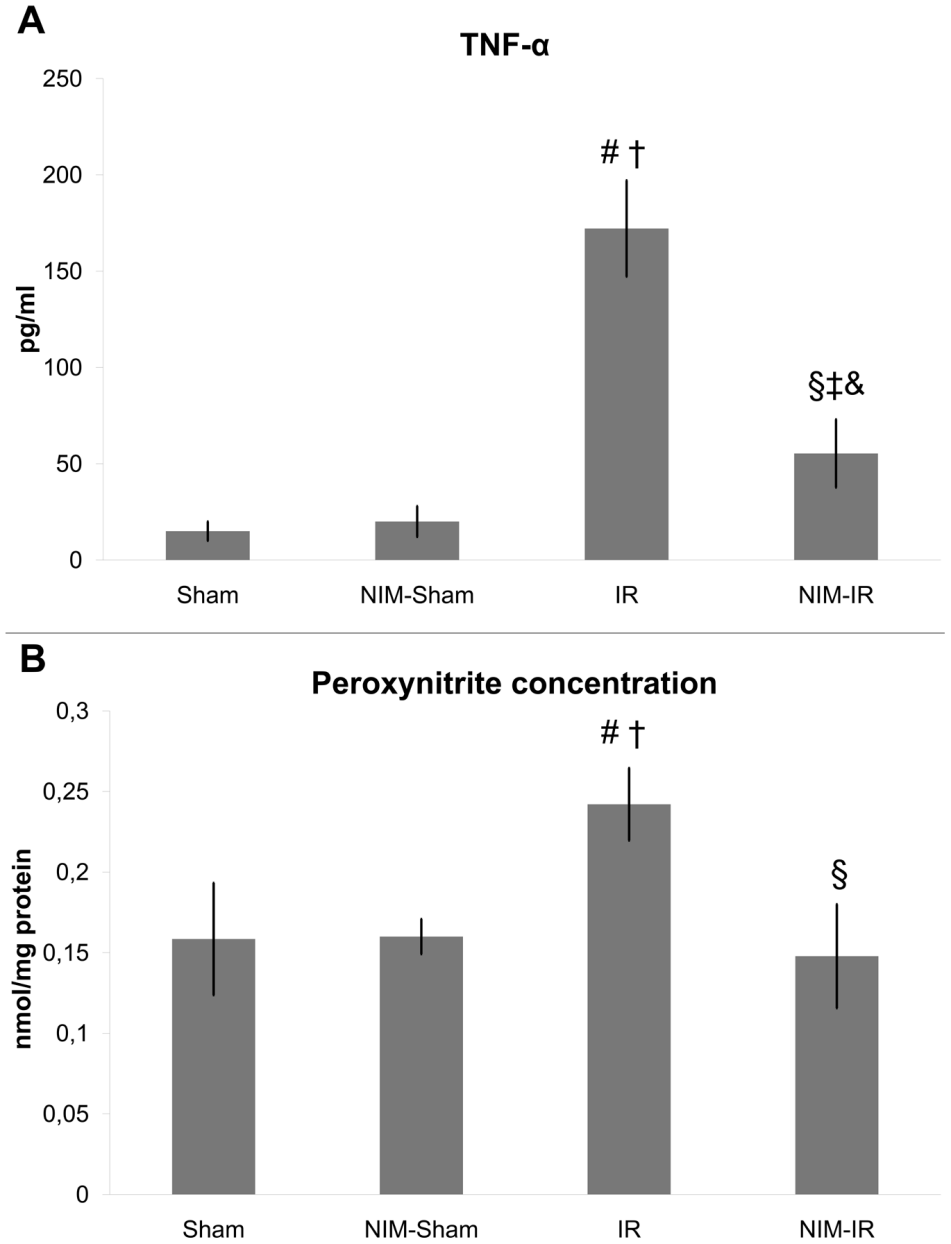
Serum TNF-α and peroxynitrite concentration in the kidney. (**A**)**:** Serum TNF-α concentration was significantly elevated in the IR group, compared with the Sham and NIM-Sham groups. A significantly lower level was detected in the NIM-IR group compared with the IR group, leading to the conclusion that there may be a lower extent of systemic inflammation (# p<0.01 vs. Sham; † p<0.01 vs. NIM-Sham; § p<0.01 vs. IR; ‡ p>0.05 vs. Sham; & p>0.05 vs. NIM-Sham; pg/ml – pictogram/mililitre). (**B**)**:** A significantly elevated peroxynitrite concentration can be observed in homogenized kidney samples in the IR group, compared with the Sham and NIM-Sham groups. In the NIM-IR group, a significantly lower value was measured compared with the IR group (# p<0.01 vs. Sham; † p<0.05 vs. NIM-Sham; § p<0.01 vs. IR).

Both TNF-α and IL-6 levels were markedly increased in the IR group in comparison with the Sham and NIM-Sham groups. The TNF-α level was considerably lower in the NIM-IR group than in the IR group (p = 0.003) (Figure 4A, Table 1). Regarding IL-6 measurements, significantly lower levels were noticeable in the NIM-IR group than in the IR group (p = 0.040). (Table 1).

### Determination of kidney dysfunction

#### Histopathology

In the IR group, swollen tubular cells, intracellular vacuolization, disintegrated necrotic cells and blurred cell borders were observable, as compared with the Sham and NIM-Sham groups. The morphological picture of the kidney was more favourable in the NIM-IR group, cell necrosis could rarely be seen, intact cell borders and nearly normal cell morphology were present. (Figure 1 E–H).

The applied histopathological score was significantly lower in the NIM-IR group, as compared with the IR group (p<0.001) (Table 1).

#### Urine output and laboratory findings

**Figure 5:**
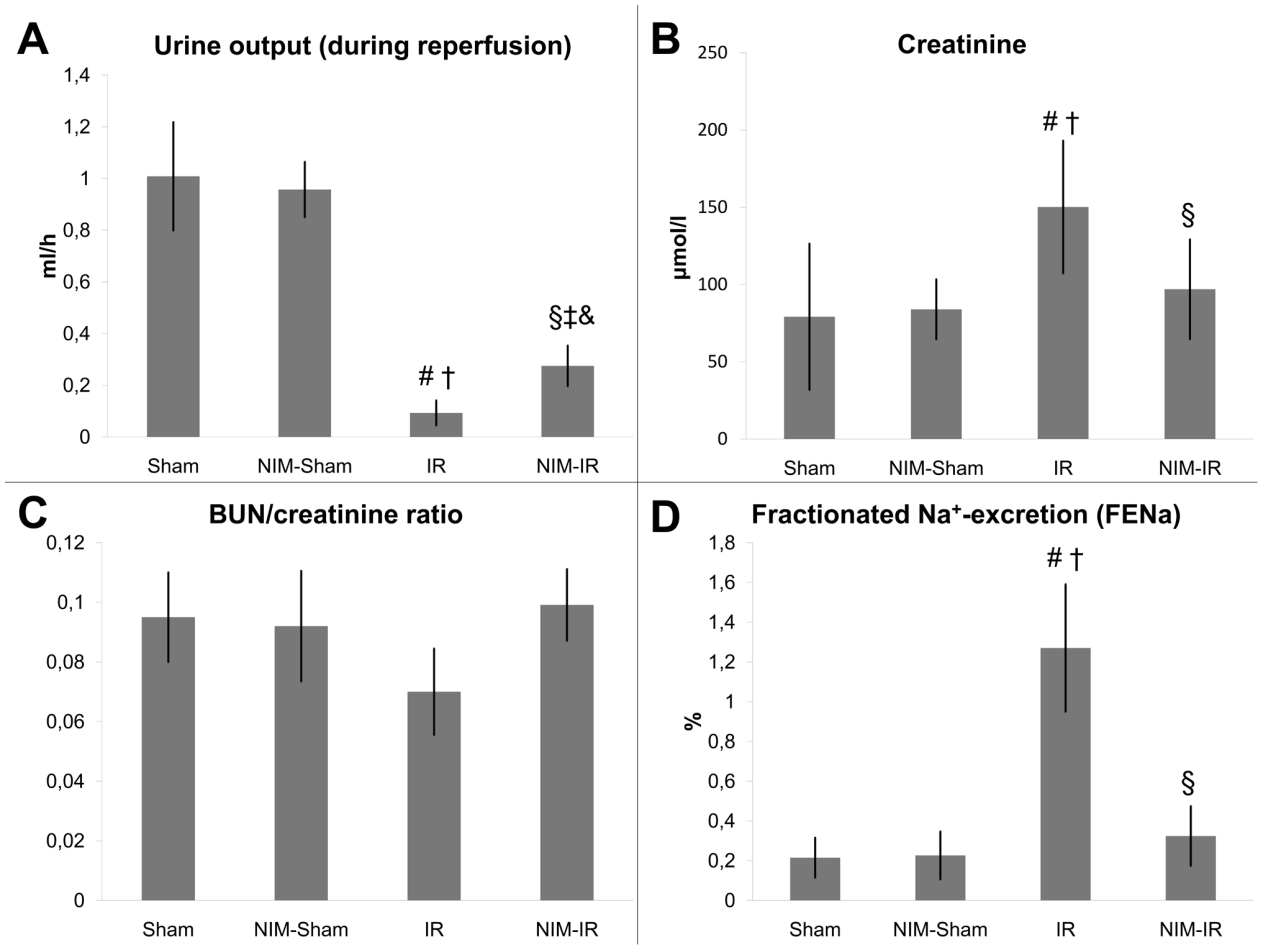
Renal function and calculated renal parameters. (**A**)**:** Based on the measured urine output during reperfusion, diuresis is significantly lower in the IR group compared to the Sham and NIM-Sham groups. In the NIM-IR group, urine output is significantly higher compared with the IR group (# p<0.01 vs. Sham; † p<0.05 vs. NIM-Sham; § p<0.05 vs. IR; ‡ p<0.01 vs. Sham; & p>0.05 vs. NIM-Sham). (**B**)**:** Serum creatinine level of the IR group is significantly elevated compared with the Sham and NIM-Sham groups. The serum creatinine level of the NIM-IR group is significantly lower compared with the IR group (# p<0.01 vs. Sham; † p<0.01 vs. NIM-Sham; § p<0.05 vs. IR). (**C**)**:** Serum BUN/creatinine ratio showed a lower value compared with the Sham and NIM-Sham groups. In the NIM-IR group, a significantly increased value was detectable than in the IR group, however the difference was not significant. (**D**)**:** The fractional Na^+^-excretion is significantly increased in the IR group compared with the Sham and NIM-Sham groups. There is a significantly lower value in the NIM-IR group than in the IR group (# p<0.01 vs. Sham; † p<0.01 vs. NIM-Sham; § p<0.01 vs. IR).

Based on the urine volume measured during the reperfusion, diuresis was significantly lower in the IR group than in the Sham and NIM-Sham groups. In the NIM-IR group, urine output was significantly higher when compared with the IR group (p = 0.022) (Figure 5A, Table 1).

The serum creatinine level observable in the IR group was significantly higher than the levels found in case of the Sham and NIM-Sham groups. The level manifest in the NIM-IR group was significantly less elevated than in case of the IR group (p = 0.001) (Figure 5B, Table 1).

#### Calculated renal functional parameters

The fractional Na^+^-excretion was significantly higher in the IR group compared with the Sham and NIM-Sham groups. A significantly lower value could be calculated in the NIM-IR group than in the IR group (p<0.001) (Figure 5D, Table 1).

The serum BUN/creatinine ratio was lower in the IR and NIM-IR groups compared with the Sham and NIM-Sham groups. No statistically significant differences could be seen between the IR and NIM-IR groups, though less decreased values were measured in the latter group (Figure 5C, Table 1).

#### Renal microcirculation

After onset of reperfusion, the microcirculatory flow rates of the IR and NIM-IR groups showed a declining tendency in comparison with the Sham and NIM-Sham groups; however, calculations of the reperfusion area (RA) values did not point to any significant differences measurable between the groups (Table 1).

#### Measurement of peroxynitrite concentrations in the kidney

Significantly higher concentration of peroxynitrite could be observed in the IR group when compared with the Sham and NIM-Sham groups. In case of the NIM-IR group, such elevation was preventable by administering NIM-811 (p<0.001) (Figure 4B, Table 1).

## Discussion

Acute limb ischemia is a sudden onset decline in the perfusion of the limb, which is a serious concern regarding limb function and even patient survival [24]. The aim of the present study was to set up an experimental model and search for a therapeutic option suitable for treatment of the postoperative complications of this vascular disease. Several pharmacological interventions [25], [26] and surgical conditioning techniques [14], [27] may be suitable approaches to mitigate the IR injuries of the skeletal muscle [28].

CsA was widely investigated in previous studies during ischemia-reperfusion [12]. In addition we can find papers in the literature pertaining to the human usage of this drug in clinical trials [29], but not in the clinical practice. The main problem with this drug is its wide range adverse effects, as immunosuppression, nephrotoxicity and hepatotoxicity. For this reason it is important to find a medication which can prevent ischemic-reperfusion injury without any severe adverse effects and additionally its usage is simple, quick, effective and specific. Therein lies one of the main novelties of our experiment.

The present study investigated the effects of the mPTP inhibitor, NIM-811 after three hours of lower limb ischemia and four hours of reperfusion in a rat model. MPTP opening has a pivotal role in the development of IR injuries, triggering cell death [30], [31]. NIM-811 is a non-immunosuppressant derivative of CsA, which is able to inhibit the opening of mPTP at the onset of reperfusion, similarly to the effects of CsA [12]. Previous studies [25], [32]–[36] have shown that NIM-811 can reduce infarct size after myocardial IR. Argaud et al. found that during 30 minutes of ligation and 4 hours of reperfusion of the circumflex coronary artery, CsA and NIM-811 pretreatment could significantly decrease the extension of the necrotic area [25]. Lim et al. investigated the effects of CsA in mice myocardial ischemic-reperfusion model. They found that i.v. CsA treatment was capable of reducing infarct size compared with placebo treated controls following IR [37]. The favourable effects of CsA have been successfully demonstrated in previous IR models [32]–[35] as well as following lower limb IR [36]. Further investigations have confirmed that both CsA and NIM-811 are able to attenuate injuries of the skeletal muscle [26], [38]. NIM-811 has not yet been investigated in regard to its effects on the systemic and distant organ impacts of these vascular diseases. Upon reviewing the literature, no studies were found which investigate the effects of this drug during lower limb skeletal muscle ischemia-reperfusion, however rhabdomyolysis and its remote organ complications are very common following vascular surgery of the lower limb arteries. The present study is therefore quite novel in this field of vascular surgical research. Moreover, mortality within 1 year following revascularisation of acute limb ischemia may be 15–20 per cent [39], which emphasizes the importance of our experiment.

There are only two publications in the literature so far reporting that NIM-811 is effective during skeletal muscle IR after autogen transplantation and replantation [26], [38]. Our results support this finding concerning the skeletal muscle. In the histological sections of the IR group, apparent necrosis, muscle fiber disintegration and edema were detectable in the muscles upon comparison with the nearly normal muscle morphology of the NIM-IR group. Our histological pictures are well supported by quantitatively with our muscle fiber viability results. Nevertheless, Cheng et al. examined the effect of NIM-811 in a rat small intestine IR model and in agreement with our skeletal muscle findings, they found NIM-811 effective in attenuating intestinal IR injury. [40] During a three hour long period of exclusion, rhabdomyolysis may develop, with the release of different metabolites, necroenzymes and K^+^ into the systemic circulation. Our study demonstrated that NIM-811 was capable of reducing the serum levels of necroenzymes and ions, which could be attributed to the lower extent of fiber disintegration possibly as the result of the inhibitory effect of NIM-811 on the opening of mPTP. Our observations are consistent with previous data, namely that both LDH level and rhabdomyolysis could be significantly attenuated by different conditioning techniques (preconditioning, postconditioning), as well as by pretreatment with CsA and NIM-811 in an experimental model of skeletal muscle IR [26].

During IR injury of the skeletal muscle, there is an increase in vascular permeability, with the development of escalating interstitial edema as a consequence of the extravascular presence of plasma proteins [41]. Upon the effect of NIM-811 treatment, significantly lower tissue wet content (edema) was measurable as compared with the IR group. Our observations are in good concordance with several other reports on the effects of CsA on interstitial edema content of the skeletal muscle. Troitzsch et al. studied the effects of CsA in an ischemia-reperfusion model of rabbits. Ischemic period was 4 hours, followed by 2 hours of reperfusion. The results of skeletal muscle W/D ratio supported the decreased edema formation in the CsA treated group. [42].

Mitochondria play a central role in the pathophysiology of IR injury, therefore NADH-tetrazolium enzyme histochemistry was performed in our study for the more accurate detection of subcellular muscle injury. This reaction is suitable for analysis of the intactness of mitochondria and the viability of muscle fibers [43]. With the use of NADH-tetrazolium enzyme histochemistry, Mowlavi et al. demonstrated that CsA can induce substantially less decrease in muscle fiber viability in a rat IR model compared with a nontreated control group [44]. This result is also consistent with our findings, namely, the NADH-tetrazolium reductase reaction of active mitochondria was significantly higher in the NIM-IR group. As NIM-811 is a specific drug for inhibition of mitochondrial mPTPs, it can prevent the potentially fateful pore opening at onset of reperfusion [25]. Inhibition of the pores can prevent the changes in high-energy phosphate metabolism and mitochondrial dysfunction [45], thereby the number of viable mitochondria remains high. A previous study by Troitzsch et al. also indicated that CsA treatment was capable of preserving the tissue viability following skeletal muscle ischemia-reperfusion according to the performed mitochondrial viability index [42].

In our experiment, we also studied the changes in microcirculation of the rectus femoris muscle. During reperfusion, a considerably higher flow was manifest in the NIM-811 treated group than in the IR group. The conservation of cell structure integrity may be in the background of this finding. Maintained integrity of the cell structure results in lower degree of muscle cell necrosis and tissue inflammation. As a consequence, less interstitial wet content is present, which contributes to the mitigation of microvascular external compression, thereby the appearance of the “no-reflow” phenomenon is less likely [46]. The integrity of microcirculation is of great importance as regards survival of the limb during lower limb IR. Microcirculation is completely stopped as the effect of total ischemia. It has been reported that after 4 hours of reperfusion, the capillary flow of skeletal muscle tissues is decreased by 50 percent [47]. Rieber et al. demonstrated the positive effect of CsA on the microcirculation of human ischemic heart following transplantation. This drug was capable of preserving of microvasculature response which has impact on long-term graft function after heart transplantation. [48].

In the background of the evolving systemic complications following lower limb IR, generalization of the local inflammatory process (SIRS) may play an important role [49]. The significant changes taking place in the systemic circulation after a 3 hour long period of exclusion underline the development of SIRS both in the IR and NIM-IR groups. Different inflammatory mediators and proinflammatory cytokines are released during the process. In our experiment, TNF-α and IL-6 showed significantly lower values in the NIM-IR group. The data of Squadrito et al. are in good concordance with our results. These authors investigated the effect of CsA after myocardial ischemia with the finding that it was able to decrease the systemic TNF-α level and thereby the progression of generalized inflammatory reaction [50].

Patients suffering from rhabdomyolysis are threatened by acute kidney injury [51]. Miller et al. also demonstrated the linkage between lower limb ischemia following vascular operation and postoperative kidney injury. They supported their findings with epidemiological evidences that ischemic rhabdomyolysis of the skeletal muscle may be a major determining factor of renal failure after thoracoabdominal aortic surgery. [52] In the current experimental study, NIM-811 had primary effect on the mPTP opening of lower limb skeletal muscle fibers and thereby an indirect effect on remote kidney injury. Based on a similar principle, Cour et al. investigated the effect of NIM-811 on the kidneys in rabbits presenting post-cardiac arrest syndrome following cardiac arrest and they demonstrated that the treatment improved the short-term survival rates and kidney function [53].

Cast formation and tubular cell injury play central role in the renal complications of rhabdomyolysis after lower limb IR. In the present study, histological assessment of the kidneys demonstrated acute tubular necrosis and intracellular vacuolization in the IR group. By comparison, the NIM-IR group showed a more favourable histological picture, also supported by the results of the performed histological scoring of the kidney tissue.

Based on the results of the skeletal muscle viability and fiber necrosis assessment, a lower degree of muscle damage developed in the NIM-IR group, thus the release of metabolites and inflammatory factors into the circulation was reduced. This could be an explanation for the observed favourable histological picture of the kidney.

The question arises regarding the direct effect of NIM-811 on the mitochondria of the kidney, since this drug is used systemically, however the main part of this pathophysiological process is the skeletal muscle damage and consequently the releasing factors and molecules which have damaging effect on the kidney. Therefore the skeletal muscle should be considered as the therapeutic target. Nevertheless our results on kidney microcirculation might support that NIM-811 has no direct effect on the kidney.

Registration of the kidney microcirculation revealed diminished blood flow in both the IR and NIM-IR groups after the onset of reperfusion, which phenomenon has also been reported in the literature after rhabdomyolysis [54]. In the current experimental model, NIM-811 treatment had no effect on the microcirculatory changes in the kidney, blood flow did not differ significantly between the IR and the NIM-IR groups. The conclusion could be drawn that NIM-811 pretreatment is able to reduce kidney injury after lower limb exclusion, but the renal protective effect is not exerted directly on the kidney.

Oxidative stress and the released free-radicals have important roles in renal dysfunction following lower limb IR [54], [55]. There are two main pathways of forming free-radicals: by way of oxidative and nitrosative reactions. Peroxynitrite (ONOO^-^) has several deleterious effects on the cell [56]–[60]. In the current experiment, accumulation of ONOO^-^ in the kidney suggests intensive production of nitrogen monoxide (NO) and development of nitrosative stress. Besides measuring the formation of ONOO^-^ in our preliminary screening studies, we also investigated the level of oxidative stress by means of measuring superoxide-dismutase (SOD) and catalase (CAT) activities, as well as accumulation of hydrogen peroxide (H_2_O_2_). Under the experimental conditions of the current study, there were no significant changes in any of the above parameters after IR (these data of preliminary studies are unpublished). The unaltered SOD and CAT activities could be the consequence of the fast depletion of O_2_ **˙** ^-^ through collision with NO and the formation of an increased amount of ONOO^-^. This implies that the damage caused by O_2_ **˙** ^-^ is mostly due to the rapid accumulation of ONOO^-^ and not to the formation of H_2_O_2_. Formation of ONOO^-^ was significantly reduced by administration of NIM-811 in the NIM-IR group. This finding correlates with the results of Packer et al., who showed that peroxynitrite induces the opening of mPTP in isolated mitochondria and can lead to the disturbance of cellular Ca^2+^ homeostasis, which contributes to cell injury. CsA has been shown to prevent this process as well [61].

In the current experimental model, kidney injury after rhabdomyolysis was determined predominantly by renal causes, namely the appearance of acute tubular necrosis. Our histological results and scoring showed that NIM-811 is capable of mitigating the tubular damages. Based on these observations, we assessed the indirect efficiency of NIM-811 treatment on the renal functions. Urine output was significantly higher in the NIM-IR group and this finding is independent from the fluid intake. Standard 5 ml/bwkg/h continuous fluid infusion was given via jugular vein cannula for each animal during the whole experiment. To take a closer look at the renal function, we used calculated renal parameters. The higher BUN/creatinine ratio observed in the NIM-IR group could be explained by the lower levels of creatinine and higher values of BUN. Urea is reabsorbed by tubular cells after glomerular excretion, which does not occur when the tubular cells are injured. We could therefore assume from the higher BUN/creatinine ratio that less tubular cells were affected in the NIM-IR group.

The fractional Na^+^-excretion is another easily calcualted parameter for renal function. The majority of filtrated Na^+^ is reabsorbed by the proximal tubular cells. A higher excretion of Na^+^ in the urine represents impaired reabsorption, thus a more severe damage of the tubular cells. NIM-811 has a favourable effect on the tubular function, which is supported by the significantly lower value of fractional Na^+^-excretion detected in the NIM-IR group.

## Conclusion

Our results demonstrated the favourable effect of NIM-811 on skeletal muscle histology and necroenzyme release. Based on our observations, NIM-811 can improve the viability of mitochondria and the microcirculation and mitigates the interstitial wet content in the injured muscle tissues of the lower limb. Systemic inflammatory parameters proved the beneficial effects of this drug on the development of systemic inflammatory reactions. Our histological and laboratory evaluations of the renal function have demonstrated the positive effect of NIM-811 on kidney injuries.

NIM-811 treatment was able to prevent and reduce rhabdomyolysis of the skeletal muscle and the remote kidney complications in our experimental model of lower limb arterial occlusive diseases and IR injuries during vascular operations.
